# Supplementary material for: The interactome of intact mitochondria by cross-linking mass spectrometry provides evidence for coexisting respiratory supercomplexes
Source: Mol Cell Proteomics. 2017 Dec 8;17(2):216–32. doi: 10.1074/mcp.RA117.000470 (PMC5795388; doi:10.1074/mcp.RA117.000470)
Supplement: Supplemental Data [file supp_RA117.000470_133922_0_supp_23978_fzffwf.zip › spectra_annotation/mito_spectra_annotation/362-1-6-1-18-1.pdf]

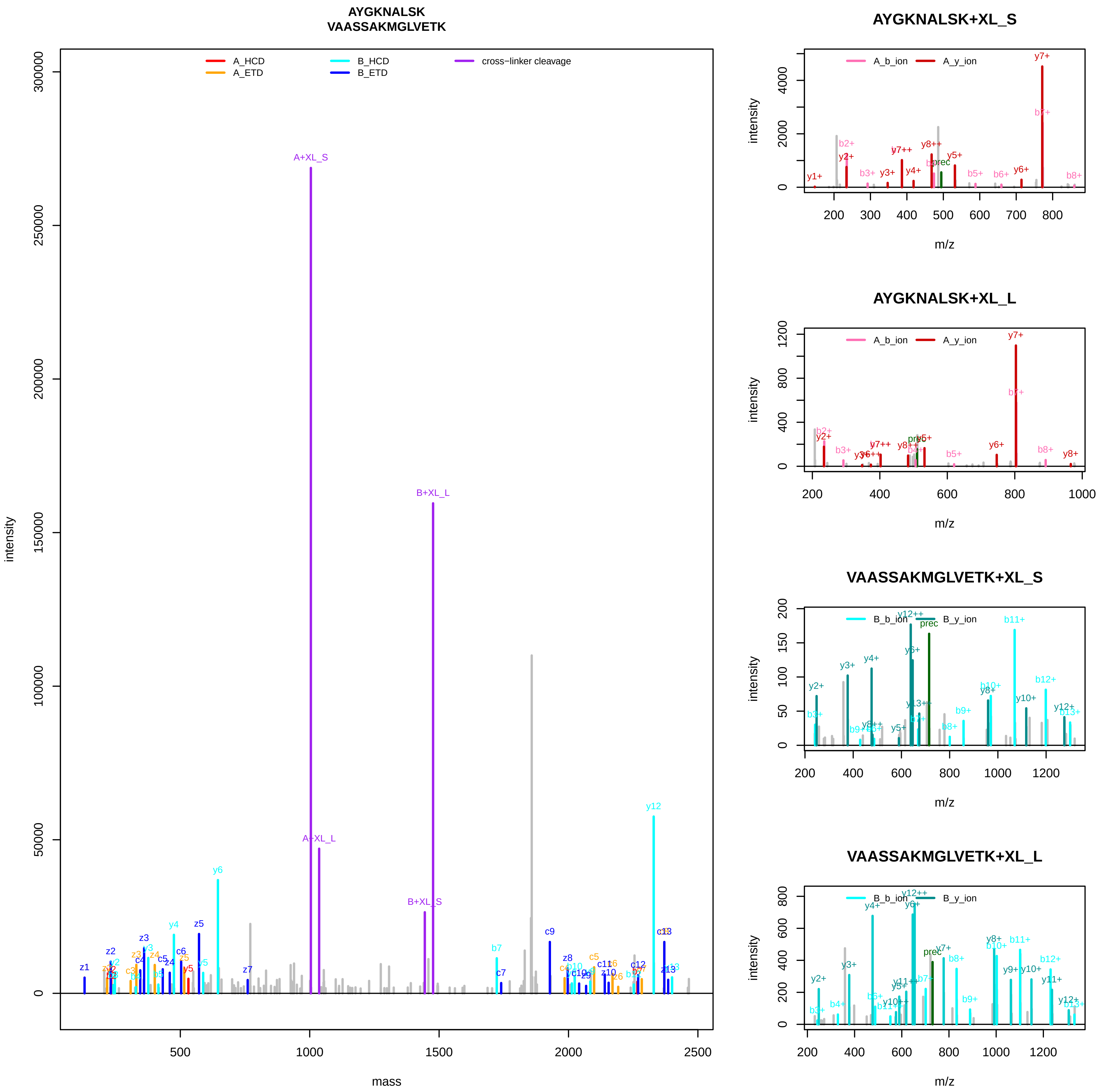

AYGKNALSK
VAASSAKMGLVETK
A_HCD
A_ETD
B_HCD
B_ETD
cross−linker cleavage
A+XL_S
B+XL_L
A+XL_L
B+XL_S
y12
y6
y4
z5
c9
c13
b7
c7
z7
z8
intensity
mass
0
50000
100000
150000
200000
250000
300000
500
1000
1500
2000
2500
AYGKNALSK+XL_S
A_b_ion
A_y_ion
y7+
b7+
b2+
y2+
y8++
y7++
y5+
prec
y1+
b3+
y3+
y4+
b5+
b6+
y6+
b8+
m/z
AYGKNALSK+XL_L
VAASSAKMGLVETK+XL_S
B_b_ion
B_y_ion
y12++
b11+
y6+
y3+
y4+
y2+
b10+
y8+
y10+
b12+
y12+
b13+
VAASSAKMGLVETK+XL_L
